# Borderline personality disorder and attention deficit/hyperactivity disorder in adolescence: overlap and differences in a clinical setting

**DOI:** 10.1186/s40479-020-00122-w

**Published:** 2020-04-15

**Authors:** Ömer Faruk Akça, Kiana Wall, Carla Sharp

**Affiliations:** 1 Meram School of Medicine, Department of Child and Adolescent Psychiatry, Necmettin Erbakan University, 42080 Konya, Turkey; 2 Department of Psychology, University of Houston, Houston, TX USA

**Keywords:** Borderline personality disorder, Attention deficit/hyperactivity disorder, Adolescent, Co-morbidity, Behavioral problems

## Abstract

**Background:**

With increased consensus regarding the validity and reliability of diagnosing Borderline Personality Disorder (BPD) in adolescents, clinicians express concern over the distinction between BPD and Attention-Deficit/Hyperactivity Disorder (ADHD), and its co-morbidity in clinical settings. The goal of this study was to evaluate differences between BPD, ADHD and BPD + ADHD in terms of co-morbid psychiatric disorders and a range of self-reported behavioral problems in adolescents.

**Methods:**

Our sample consisted of *N* = 550 inpatient adolescents with behavioral and emotional disorders that have not responded to prior intervention. We took a person-centered approach (for increase clinical relevance) and compared adolescents with ADHD, BPD and ADHD+BPD in terms of co-occurring psychiatric disorders and behavioral problems. We performed a regression analysis to test whether BPD symptoms make an incremental contribution to the prediction of psychiatric symptoms over ADHD symptoms.

**Results:**

The severity of almost all co-occurring disorders, aggression, self-harm, suicidal thoughts, and substance use, were higher in the ADHD+BPD group. Borderline symptoms made an incremental contribution to the prediction of psychiatric symptoms beyond the contribution of ADHD.

**Conclusion:**

Severity and co-morbidity may be helpful factors in distinguishing between ADHD and BPD in clinical practice and the co-morbidity of these two disorders may indicate a worse clinical outcome.

## Background

Borderline Personality Disorder (BPD) is a complex and severe mental disorder which is characterized by impulsivity, affect dysregulation and dysfunctional interpersonal relationships. Individuals with BPD demonstrate pervasive patterns of unstable interpersonal relationships, pronounced impulsive and self-damaging- behavior, unstable identity and difficulties in emotion regulation [1] which substantially worsen psychosocial, occupational and educational functioning [2, 3]. Increasing evidence indicates that BPD features are present in childhood and adolescence, and that BPD can be reliably diagnosed in adolescence [3–8]. Evidence has also been accumulating to suggest that BPD can benefit from early intervention [9–11].

With increased consensus regarding the validity and reliability of diagnosing Borderline Personality Disorder (BPD) in adolescents, clinicians express concern over the distinction between BPD and Attention-Deficit/Hyperactivity Disorder (ADHD) in clinical settings. Attention Deficit and Hyperactivity Disorder (ADHD) is a neurodevelopmental disorder characterized by inattention, hyperactivity, and impulsivity in addition to problems in organization and staying focused [12]. These are features that overlap with the impulsivity and emotion dysregulation criteria of BPD. Indeed, longitudinal studies show that ADHD diagnoses in childhood are associated with the development of Personality Disorders in adulthood [13]. Furthermore, adults with ADHD have higher co-morbidity of Personality Disorders, especially Avoidant, Narcissistic, Paranoid, Antisocial and Borderline Personality Disorders [14–16]. In addition, Philipsen et al. found that 41% of adults with BPD reported high rates of ADHD in childhood. Additionally, the authors reported that severe BPD symptoms in adulthood were related to a diagnosis of ADHD in childhood [17]. Several follow-up studies support this relationship and provide evidence for the sequential nature of the relationship. Specifically, Miller et al. reported that 13.5% of the children with ADHD -but only 1.2% of controls- were diagnosed with BPD in adolescence. Moreover, they reported that youth who continued to meet criteria for the ADHD diagnosis in adolescence had higher rates of BPD compared to youth who remitted from ADHD [16]. Studies report that BPD and ADHD are commonly co-morbid in both adults and adolescents [9, 18]. Ferrer et al. found that in a clinical setting 38% of the patients referred with a BPD diagnosis met criteria for ADHD as well [19]. Likewise, population surveys report that 33% of subjects with ADHD also have BPD, compared to a BPD prevalence of only 5% in the general population [20], and subjects diagnosed with ADHD been found to have a very high rate of BPD compared to individuals without ADHD (odds ratio:19.4) [21].

There are multiple ways in which the high rates of co-morbidity between ADHD and BPD can be conceptualized and studied. One approach may be to use variable-centered factorial analyses to examine whether ADHD and BPD represent different clinical presentations of the same underlying construct or constellation of symptoms. Another approach may be to use variable-centered approaches in longitudinal designs to examine whether ADHD and BPD are sequentially related portions of the same construct or disorder. A third approach, which is consistent with a more traditional DSM-based medical model approach is to take a person-centered view by asking if ADHD and BPD have differential correlates when considered separately and combined [9]. This approach may represent a clinically relevant approach that can immediately be translated into clinical settings where clincians still make use of traditional DSM-based diagnostic categories when they take decisions about individual patients, as opposed to variable-centered nomothetic approaches. Taking this approach, several studies report that co-morbidity between ADHD and BPD results in worse psychosocial outcomes. In particular, individuals with both ADHD and BPD have higher rates of other psychiatric disorders (i.e. Depression, Anxiety, Obsessive-Compulsive Disorder, Disruptive Behavior Disorders, Substance Abuse, and Antisocial Personality Disorder) [19, 22, 23], greater impulsivity and aggression [24], and are more likely to have a history of abuse and neglect in their childhood compared to BPD only subjects [25]. In addition, several BPD symptoms have been found to be greater in subjects with both ADHD and BPD (ADHD+BPD) group compared to subjects with only BPD [23]. Based on these findings, researchers have suggested that patients who present with both disorders might be classified in a different group than BPD or ADHD alone [19].

However, these findings are based mostly on adult studies and the outcome of the co-occurrence of ADHD and BPD in adolescence (the time period in which BPD symptoms are likely to emerge) is not well studied. In addition, previous studies of adults with ADHD and BPD have largely conducted purely categorical comparisons of the disorders and none of these studies were designed to investigate the relationship between ADHD and BPD symptoms from a symptom-based viewpoint. To our knowledge, only one study has been conducted on this topic in adolescents. With a relatively small clinical sample, Speranza et al. compared BPD and ADHD+BPD adolescents in terms of their traditional Axis I and II diagnoses. Nine (11%) of the 85 BPD adolescents received an additional ADHD diagnosis, and this ADHD+BPD group evidence higher incidence of Disruptive Behavior Disorder diagnosis compared to the BPD only group [23]. Apart from the relatively low sample size, as an additional limitation, this study did not include an ADHD-only group to provide the possibility to compare ADHD with ADHD+BPD subjects. In addition to DSM IV diagnoses, they did not include any other variables of interest -except for impulsivity- to compare the groups. However, these two disorders are well known to be related to several behavioral problems like substance use, aggression, suicidality, and self-harm [1, 26].

Taking into account the gap in the literature on this subject in adolescents, the aims of the current study were twofold. First, we aimed to compare inpatient adolescents who received a diagnosis of ADHD, BPD or both (ADHD + BPD) diagnoses in terms of their psychiatric disorder severities determined with a semi-structured interview, and self-reported behavioral problems. We hypothesized that adolescents with both disorders would demonstrate a greater number of symptoms of DSM based traditional Axis I diagnoses and other behavioral problems (i.e. substance use, aggression, suicidal ideation, and self-harm) compared to subjects with only ADHD or BPD. In addition, based on previously discussed literature reporting that severe BPD symptoms in adulthood are related to a diagnosis of ADHD in childhood [17], we hypothesized that the ADHD+BPD group would demonstrate higher BPD symptoms compared to BPD-only group. Our second aim was to test whether BPD symptoms had an incremental contribution over and above ADHD symptoms in predicting total psychiatric symptomatology in inpatients, to contribute to debates on whether BPD and ADHD are the same or different disorders. Despite broad agreement that these two disorders are different constructs, [14] this assumption is mostly based on adult studies. The relation of the two disorders to overall psychiatric symptomatology has not been explored in adolescent samples. During adolescence, many psychiatric disorders onset and it may be that it is more difficult to discriminate between the BPD and ADHD in adolescence compared to in adulthood. We hypothesized that BPD symptoms would make an incremental contribution to the prediction of total symptomatology beyond the ADHD symptoms of the patients, thereby demonstrating discriminatory associations with outcomes.

## Methods

### Participants and procedures

Participants in the current sample were recruited between October of 2008 and June of 2016 from an inpatient psychiatric hospital located in the United States which serves adolescents with behavioral and emotional disorders that have not responded to prior intervention. All procedures in the current study were approved by the appropriate human subjects review committee. Parents of admitted adolescents were approached and invited to participate in the study. If parents consented, adolescents were approached to provide assent. Adolescents had to demonstrate sufficient proficiency in English to provide assent and to complete study assessments, and they were excluded from participation if they had a diagnosis of schizophrenia or other psychotic disorders, bipolar disorder, autism spectrum disorder or an IQ < 70. Study assessments were administered by trained research coordinators and/or doctoral-level clinical psychology students. Assessments were conducted individually and in private within the first 2 weeks following admission.

Of *N* = 805 consecutively admitted adolescents and their parents, *n* = 52 declined participation, *n* = 117 were excluded from participation based on study criteria and *n* = 86 were excluded because of absent data on measures utilized in the current study. The final sample consisted of *n* = 550 adolescents (63% female; ages 12–17, M = 15.37, SD = 1.43), with the following racial/ethnic breakdown: 77.6% White (*n* = 427), 5.8% multiracial or other (*n* = 32), 3.3% Asian (*n* = 18), 1.6% Black or African American (*n* = 9), 0.2% American Indian or Alaskan Native (*n* = 1) and 11.5% unspecified (*n* = 63). Based on the Diagnostic Interview Schedule for Children – Computerized Version (DISC-IV) [27] conducted with adolescents at admission, 52% of the sample met criteria for an anxiety disorder, 50.3% met criteria for a depressive disorder, 36.8% met criteria for an externalizing disorder, 9.1% met criteria for a substance use disorder, and 7.7% met criteria for an eating disorder.

**Fig. 1:**
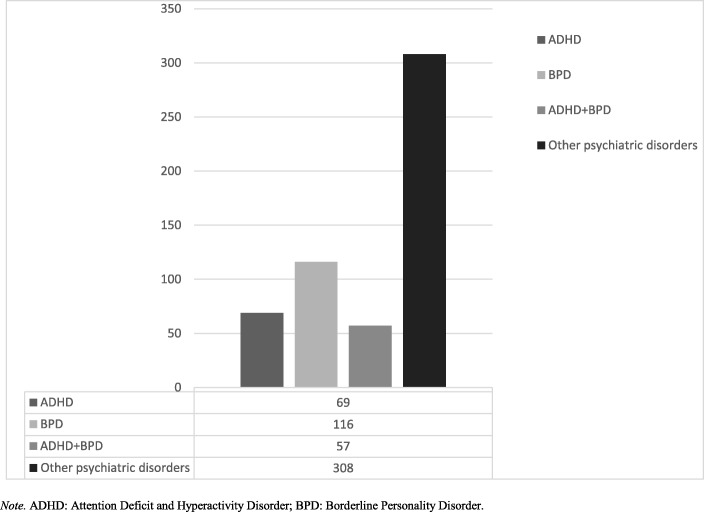
Diagnostic distribution of the subjects who participated in the study. *Note.* ADHD: Attention Deficit and Hyperactivity Disorder; BPD: Borderline Personality Disorder

Among the patients who participated in the study, 69 (12.5%) received a diagnosis of ADHD without BPD, 116 (21.1%) received a diagnosis of BPD without ADHD and 57 (10.3%) received both ADHD and BPD diagnosis (see Fig. 1). The prevalence of ADHD and BPD seems high in our study though, however, it should be kept in mind that our sample was consisted of adolescents who did not respond to prior intervention as we have mentioned previously. In addition, several previous studies report high rates of BPD in inpatient sample consistent with our finding [28, 29]. Thirty three percent of the patients diagnosed with BPD received an additional ADHD diagnosis, and 45% of the patients with ADHD received an additional BPD diagnosis. Mean ages of the ADHD, BPD and ADHD+BPD groups were 15.1, 15.5 and 15.1 respectively and there was not a significant difference between groups in terms of age (*p* = 0.19). However, the gender distribution of the groups were different (female ratio of the ADHD, BPD and ADHD+BPD groups were 18, 54 and 27% respectively, *p* < 0.001) (see Table 1).

**Table 1:** Comparison of the ADHD, BPD and ADHD+BPD groups in terms of age and gender

	ADHD	BPD	ADHD+BPD	Total	p
Female n (%)	33 (18.7)	95 (54.0)	48 (27.3)	176 (100)	< 0.001
Male n (%)	36 (54.6)	21 (31.8)	9 (13.6)	66 (100)	
Age (Mean ± SD)	15.1 ± 1.5	15.5 ± 1.5	15.1 ± 1.5		0.19

### Measures

*The Diagnostic Interview Schedule for Children – Computerized Version (DISC-IV)* [27] is a structured clinical interview assessing DSM IV Axis 1 diagnoses and is designed for use with children and adolescents ages 9–17. The DSIC-IV youth interview has demonstrated adequate validity and test-retest reliability in community and clinical samples of youth [27]**.** Results of the DISC-IV are categorically coded to indicate the presence or absence of each disorder: 0 = no diagnosis, 1 = intermediate diagnosis, 2 = positive diagnosis. In the current study, the DISC-IV was utilized to determine ADHD diagnostic status of the participants and the overall number of diagnoses each adolescent received. In addition, the DISC-IV also reports the total number of symptoms endorsed by adolescents for each disorder. In the current study, total number of reported symptoms (including anxiety disorders, eating disorders, Major Depressive Disorder, Conduct Disorder, Oppositional Defiant Disorder, Obsessive Compulsive Disorder, Post-Traumatic Stress Disorder and Substance Abuse Disorder) were conceptualized as the severity criterion for each disorder and used as a continuous outcome variable in analyses. Also, total number of all reported symptoms were used in the current study as a severity variable to evaluate overall symptomatology. To avoid confusion, the name of the disorders analyzed in this study (except for description analyses of the groups) will indicate their symptom severity in the remaining part of the article (i.e. Major Depressive disorder, eating disorders etc.).

*The Childhood Interview for DSM-IV Borderline Personality Disorder (CI-BPD* [30]; is a semi-structured interview, adapted from the Diagnostic Interview for Personality Disorders [31], to assess BPD in youth. The interview is divided into 9 sections reflecting diagnostic criteria for BPD and each section is rated on a 0–2 scale by the interviewer (0 – symptom is absent; 1 – symptom probably present; 2 – symptom definitely present). A diagnosis of BPD requires that at least five criteria be rated a “2”. The CI-BPD has demonstrated good internal consistency (α = .80) and excellent interrater reliability (κ = .89) in prior studies of adolescent inpatients [32]. In the current sample, 3-way agreement (κ = .609, *p* < .0005) between raters on item 10 of the CI-BPD (0 – BPD absent; 1 – subthreshold for BPD criteria; 2 – meets five or more BPD criteria) and two-way agreement (κ = .739, *p* < .0005) between raters (0 – BPD absent or sub-threshold; 1 – BPD present) was good. In the current study, the interview’s dichotomous rating (0 – BPD absent or sub-threshold; 1 – BPD present) was utilized to determine BPD diagnostic status and a continuous score (each item scored 0–2 and summed) was utilized for group comparisons and regression analyses.

*The Personality Assessment Inventory for Adolescents (PAI-A)* [33] is a self-report measure of personality and psychopathology which consists of 264 items rated on a 4-point Likert scale ranging from “not at all true” to “very true”. The PAI-A yields raw scale scores and also T-scores derived from standardization with census-matched community samples and a clinical sample of outpatient youth. The PAI-A has demonstrated good internal consistency, test-retest reliability and construct validity in both clinical and community samples of youth [33]. In the current study, only the T-scores of the alcohol problems (ALC), drug problems (DRG), suicidal ideation (SUI), aggression (AGG) and borderline-self-harm (BOR-S) subscales were used. The ALC and DRG subscales are clinical scales which measure an individuals’ difficulty with excessive drinking and recreational drug use, respectively. The BOR-S measures self-injurious behavior and is a subscale of the Borderline Features Clinical Scale. The suicidal ideation and aggression subscales are treatment consideration scales. They aim to measure features of an individual’s personality or presentation which are relevant to treatment and may serve as risk factors for psychopathology but may not be measured as part of a psychiatric diagnosis. The suicidal ideation subscale measures the frequency and severity of an individual’s suicidal thoughts, plans and actions. The aggression subscale measures their aggressive behavior towards others.

### Data analytic strategy

Statistical analyses were performed using SPSS 20.0 statistical software (SPSS Inc., Chicago, IL, USA). All variables were inspected for normality with the Kolmogorov-Smirnov test. First, the sample was divided into three groups based on the diagnostic results of the DISC-IV and the CI-BPD. The first group consisted of adolescents who met diagnostic criteria for ADHD and not for BPD. The second group consisted of adolescents who met diagnostic criteria for BPD and not for ADHD. The third group consisted of adolescents who met diagnostic criteria for both ADHD and BPD. To test our first hypothesis, Kruskal Wallis analysis was conducted to determine whether significant group differences existed among the aforementioned groups in terms of the number of diagnoses they received on the DISC-IV, the total number of symptoms they endorsed for each diagnosis on the DISC-IV (except for ADHD and BPD), their total scores on the CI-BPD and their T-scores on the PAI-A ALC, DRG, SUI, AGG and BOR-S subscales. The between-group analyses were conducted using Mann Whitney U test performing a post-hoc Bonferroni correction. Finally, a hierarchical regression model was conducted on the full inpatient sample (regardless of their diagnostic group) to determine whether number of BPD symptoms had incremental predictive validity over total number of ADHD symptoms in predicting overall psychopathology –our second hypothesis-, operationalized as total number of symptoms endorsed on the DISC-IV. At the first step, we have tested the predictive value of ADHD, age and gender on total psychiatric symptoms determined with DISC-IV. Then, we have added the total number of BPD symptoms of the participants to the model at the second step.

## Results

### BPD vs. ADHD vs. BPD + ADHD group comparisons on psychiatric symptoms and behavioral problems

We tested our first hypothesis with comparison analyses and found that the groups were significantly different in terms of all psychiatric symptoms and behavioral problems except for Substance Abuse, Conduct Disorder and Obsessive Compulsive Disorder symptoms (Tables 2 and 3). Post-hoc analyses showed that the ADHD+BPD group showed greater self-reported behavioral problems and interview-based psychiatric symptoms, for almost all disorders, than the ADHD or BPD-only groups. Likewise, the ADHD+BPD group received more psychiatric diagnoses than the ADHD and BPD groups. Post-hoc analyses conducted between the ADHD and BPD groups showed that Eating Disorders, Depression symptoms, internalizing disorders and suicidal ideation were higher in the BPD group compared to the ADHD group, however, only the total number of externalizing disorder symptoms was greater in the ADHD group than the BPD group.

**Table 2:** Comparison of the ADHD, BPD and ADHD+BPD groups in terms of number of co-morbid DSM -IV diagnoses, clinical severities of co-morbid disorders and borderline personality disorder measured by interviews

DSM IV Symptoms (based on DISC-IV)	ADHD (1)	BPD (2)	ADHD+BPD (3)	*X*^*2*^	p	Post hoc (adjusted *p* = 0.017)
Total number of diagnoses	2.3 ± 2.2	2.7 ± 2.0	3.8 ± 2.4	15.6	< 0.001	3 > 1, 3 > 2
Anxiety disorders	17.0 ± 11.2	19.8 ± 10.4	20.7 ± 9.8	6.6	0.038	3 > 1
Depression	13.6 ± 4.4	15.2 ± 3.9	16.6 ± 3.4	18.7	< 0.001	3 > 2 > 1
ADHD	13.1 ± 3.6	7.9 ± 5.4	14.5 ± 3.6	71.2	< 0.001	3 > 2, 1 > 2
Oppositional Defiant Disorder	5.8 ± 3.0	6.2 ± 3.1	8.5 ± 2.7	31.4	< 0.001	3 > 1, 3 > 2
Conduct Disorder	4.5 ± 4.1	5.2 ± 4.4	6.4 ± 5.2	4.8	0.09	
Post Traumatic Stres Disorder	1.8 ± 4.0	3.3 ± 5.2	4.4 ± 6.0	7.6	0.023	3 > 1
Obsessive Compulsive Disorder	1.3 ± 1.8	1.3 ± 1.6	1.3 ± 1.4	1.1	0.57	
Eating Disorders	2.1 ± 1.9	3.7 ± 2.4	4.1 ± 2.4	25.5	< 0.001	3 > 1,2 > 1
Substance Abuse Disorder	0.7 ± 2.1	1.1 ± 3.5	0.7 ± 2.5	0.2	0.91	
Internalizing disorders	36.0 ± 16.7	43.3 ± 15.9	47.1 ± 17.2	17.4	< 0.001	3 > 1,2 > 1
Externalizing disorders	23.9 ± 8.9	19.8 ± 12.3	29.9 ± 9.5	40.9	< 0.001	3 > 1 > 2
**CIBPD**	6.8 ± 3.4	13.7 ± 2.0	14.4 ± 2.0	142.6	< 0.001	3 > 2, 3 > 1

*ADHD* attention deficit/hyperactivity disorder, *BPD* borderline personality disorder, *CIBPD* childhood interview for borderline personality disorder, *DISC-IV* the diagnostic interview schedule for children – Computerized Version

**Table 3:** Comparison of the ADHD, BPD and ADHD+BPD groups in terms of behavioral problems assessed with self-report measures (PAI-A)

Behavioral Problems	ADHD (1)	BPD (2)	ADHD+BPD (3)	*X*^*2*^	p	Post hoc (adjusted p = 0.017)
Alcohol problems	53.1 ± 15.7	53.8 ± 18.0	59.6 ± 18.6	7.5	0.023	3 > 2
Drug problems	60.0 ± 19.8	60.3 ± 19.2	68.4 ± 21.5	7.5	0.023	3 > 2, 3 > 1
Aggression	51.3 ± 11.6	54.4 ± 11.6	60.8 ± 11.7	18.4	< 0.001	3 > 2,3 > 1
Suicidal ideation	66.3 ± 18.9	76.7 ± 18.8	80.0 ± 16.3	16.8	< 0.001	3 > 1, 2 > 1
Borderline self harm	60.9 ± 13.1	64.6 ± 14.9	74.1 ± 12.3	25.8	< 0.001	3 > 2, 3 > 1

*ADHD* attention deficit/hyperactivity disorder, *BPD* borderline personality disorder, *PAI-A* the personality assessment inventory for adolescents

### Incremental value of BPD in predicting general psychiatric severity over and above ADHD

Finally, to test our second hypothesis, we conducted a hierarchical regression analysis showing that gender and ADHD symptoms -at the first step- predicted total psychiatric severity measured by DISC-IV (symptom count) (R square = 0.27, *p* < 0.001). When we added BPD symptoms -at the second step-, we found that adding BPD symptoms to the model resulted in statistically significant incremental validity over ADHD symptoms on total psychiatric symptoms (R square = 0.38, R square change = 0.11, p < 0.001) (see Table 4).

**Table 4:** Hierarchical regression analysis predicting total psychiatric symptoms as an index of general psychiatric severity

Model		B	Std. Error	Beta	t	p
1	(Constant)	50.269	12.798		3.928	< 0.001
	ADHD	2.046	0.224	0.507	9.135	**< 0.001**
	Gender	−6.729	2.735	−0.138	−2.460	**0.015**
	Age	−0.360	0.821	−0.025	−0.439	0.661
2	(Constant)	29.490	12.207		2.416	0.016
	ADHD	2.246	0.209	0.556	10.767	**< 0.001**
	Gender	−0.961	2.668	−0.020	− 0.360	0.719
	Age	−0.743	0.758	−0.051	−0.980	0.328
	CIBPD	1.925	0.293	0.360	6.574	**< 0.001**

*ADHD* attention deficit/hyperactivity disorder, *CIBPD* childhood interview for borderline personality disorder

## Discussion

Against the background of clinician concern over the distinction between BPD and ADHD, and its co-morbidity in clinical settings, the goal of the current study was to evaluate differences between BPD, ADHD and BPD + ADHD in terms of co-morbid psychiatric disorders and a range of self-reported behavioral problems in inpatient adolescents. Consistent with findings from adult samples, results of the current study suggest that there is high co-morbidity between ADHD and BPD (i.e. 33 % of patients diagnosed with BPD received an additional ADHD diagnosis, and 45% of the patients with ADHD received an additional BPD diagnosis) and that patients who meet the diagnostic criteria of both disorders demonstrate greater symptoms of numerous interview-based psychiatric disorders (*X*^*2*^:15.6, *p* < 0.001) and self-reported behavioral problems. For example, alcohol (*X*^*2*^:7.5, *p* = 0.02) and drug problems (*X*^*2*^:7.5, p = 0.02), aggression (*X*^*2*^:18.4, p < 0.001), suicidal ideation (*X*^*2*^:16.8, p < 0.001) and self-harm scores (*X*^*2*^:25.8, p < 0.001) were higher in the combined group than in the ADHD or BPD groups. Results of the hierarchical regression analysis furthermore suggested that, despite the overlap between BPD and ADHD, BPD symptoms have an incremental contribution to total psychiatric symptoms beyond ADHD symptoms, suggesting some unique features of each disorders in addition to overlap. Our sample showed that ADHD and BPD are highly associated with each other, as reported in several previous studies. However, very most studies conducted in adolescents on this topic reported lower rates of co-occurrence of ADHD and BPD, compared to our findings. Korsgaard et al. investigated personality disorders in adolescents and found that only 4 out of 21 ADHD patients (19%) also received a BPD diagnosis [34]. Similarly, Speranza et al. reported that only 11% of subjects with BPD received an ADHD diagnosis as well in their clinical sample [23] compared to 33% in our sample. Numerous adult studies report higher rates similar to our findings [19]. The diverse rates reported among studies may be explained by the differences in the samples of the studies. We conducted our study in an inpatient psychiatric hospital, which admits adolescents with behavioral and emotional disorders that have not responded to prior treatment. Thus, it could be expected that many of the subjects in our sample would have multiple, severe psychiatric problems that could result in higher diagnostic co-occurrence rates. In addition to high rates of ADHD among subjects with BPD, we found that subjects with ADHD had very high rates of BPD diagnoses (45%) compared to previous reports (18%) [35]. Similarly, this result should be interpreted keeping in mind that our sample was an inpatient group. ADHD is mostly treated in outpatient clinics and ADHD subjects rarely need to be hospitalized. Thus, our results are not representative of the non-severe cases of ADHD.

It is well known that both ADHD and BPD are highly associated with other psychiatric disorders and behavioral problems [36, 37]. However, our study revealed that the ADHD+BPD group demonstrated a greater number of psychiatric symptoms and behavioral problems compared to the ADHD or BPD groups. This finding is in line with findings obtained from adult studies. For example, O’Malley et al. reported that the subjects with both ADHD and BPD demonstrated higher levels of clinical syndromes of anxiety, somatoform, dysthymia, drug dependence, PTSD and major depression compared to subjects with only ADHD [22]. Likewise, subjects with both ADHD and BPD have been shown to have higher substance abuse/dependency rates compared to ADHD or BPD subjects, however, they did not differ in terms of other traditional axis 1 diagnoses [24]. Another study reported higher rates of substance use disorder in subjects with ADHD and BPD compared to those with only BPD [19]. Since studies of adolescents on this topic are very limited, we have restricted background of information that is based on one study which reported that ADHD+BPD patients show higher rates of disruptive behaviors than only-BPD patients. However, no group differences were reported in terms of other clinical variables (i.e. mood, anxiety, eating and substance related disorders) [23].

Our sample revealed no difference among groups in terms of substance abuse symptoms evaluated by the DISC-IV interview. However, group comparisons of the alcohol and drug subscales of PAI which evaluates the severity of alcohol and drug use with self-report scales revealed significant differences among groups, indicating that frequency of substance use differs among groups rather than addictive use. Group comparisons further revealed that the ADHD+BPD group had higher self-harm scores than both BPD and ADHD subjects. Additionally, the ADHD+BPD group had higher suicidal ideation scores than ADHD group, however, the suicidal ideation scores of ADHD+BPD and BPD groups was not different. Self-harm behavior and suicide are among the characteristic features of BPD, and is also observed in ADHD patients [1, 11, 26]. However, the contribution of ADHD symptoms to BPD in terms of self-harm and suicide in adolescents is not known. One study compared adults with ADHD, adults with BPD and adults without ADHD or BPD and found that BPD subjects have higher self-harm rates than those with ADHD [38]. Similarly, a few studies have compared ADHD+BPD subjects with BPD subjects in terms of suicidal behavior and they report no significant differences among groups [19, 24]. Despite insufficient data on this topic, the current adult literature on this issue is compatible with our findings that ADHD symptoms might contribute to the presence of self-harm, but not to suicidal thoughts.

The relationships between aggression and ADHD and aggression and BPD are well documented [39, 40]. However, like the other constructs we have studied, it is not well known whether ADHD symptoms contribute to aggression severity in BPD. Two adult studies have been conducted on this topic and found that ADHD+BPD subjects demonstrate higher aggression levels than subjects with BPD or ADHD [24] and ADHD+BPD subjects have lower anger control compared to ADHD subjects [41]. In addition, several studies report that ADHD+BPD patients have higher impulsivity levels - a construct related to aggression - [42] than BPD or ADHD patients [19, 23, 43, 44]. This adult literature is also in line with our findings that both ADHD and BPD are significantly related to aggression and that subjects who have the two disorders together have higher aggression levels.

We also compared BPD and ADHD symptom severities among the groups and found that BPD symptoms are higher in the ADHD+BPD group compared to the BPD group. This finding supports the findings of previous studies conducted in adolescents demonstrating that subjects with BPD and ADHD have higher scores on several subscales of a diagnostic interview (i.e. cognition and impulsivity subscales of Revised Diagnostic Interview for Borderline) compared to BPD only subjects [23]. However, although the number of ADHD symptoms was higher in the combined group than the ADHD group, this difference was not statistically significant indicating that having BPD does not increase the severity of ADHD. O’Malley et al. reported that an ADHD+BPD group had higher ADHD symptoms than only ADHD subjects, contrary to our findings [22]. This difference in findings may be attributed to the study samples. O’Malley et al. conducted their study on an outpatient group and it is possible that is was composed of subjects with mild and uncomplicated psychiatric symptoms compared to our inpatient sample.

These comparison analyses demonstrated that the patients in the ADHD+BPD group had higher levels of psychiatric symptoms, behavioral problems and aggression than the subjects with BPD only. Therefore, our findings support the previous adult studies claiming that a combination of BPD and ADHD is a more complicated clinical presentation which could contribute to difficulties in the treatment process and conclude in worse outcomes, and may be a different sub group of BPD [19].

Our finding that BPD symptoms demonstrated an incremental contribution to the prediction of total psychiatric symptomatology suggests that BPD symptoms are related to several psychiatric symptoms regardless of ADHD symptoms supporting the hypotheses that, although they might share several similar developmental pathways and clinical presentations, ADHD and BPD may contribute unique features in the outcomes of patients’ lives [14, 45]. It is important, however, that the overlap between these disorders are better understood and that shared endophenotypes also in youth be investigated.

The findings of this study should be considered in light of several limitations. We conducted our study in a psychiatric inpatient sample. However, as we have mentioned before, many patients with ADHD and BPD do not need to be treated by hospitalization unless their clinical appearance is complicated and severe. Also, although adolescents were evaluated at admission we did not consider their previous treatments including previously prescribed medications. Furthermore, although most of our data were obtained via semi-structured interviews, some was obtained via self-report (i.e. PAI-A), although we consider the multiple method approach in our study a strength. Also, our study did not include a healthy control group to compare the variables among the clinical and healthy groups. Finally, given a general move towards dimensional assessment of latent constructs in psychiatry and clinical psychology, our person-centered approach may be viewed as out of step with current trends. However, we aimed to provide information that can readily used by clinicians when they assess for ADHD and BPD using traditional psychiatric nosology which is still very much in place in clinical settings.

## Conclusion

The findings of this study demonstrated that ADHD and BPD have different psychiatric symptomatology. In addition, subjects who meet criteria for both the BPD and ADHD diagnoses may have more severe psychiatric and behavioral problems compared to individuals with only ADHD or BPD. We believe that these findings could help clinicians to better understand adolescents with ADHD and BPD in the diagnosis and treatment processes.

## Data Availability

The datasets of the current study are available from Dr. Sharp on reasonable request.

## Abbreviations

BPD: Borderline personality disorder
ADHD: Attention-deficit/hyperactivity disorder
DISC-IV: Diagnostic interview schedule for children – computerized version
PAI-A: Personality assessment inventory for adolescents
ALC: Alcohol problems
DRG: Drug problems
SUI: Suicidal ideation
AGG: Aggression
BOR-S: Borderline-self-harm
CIBPD: Childhood interview for DSM-IV borderline personality disorder
DSM-IV: Diagnostic and statistical manual of mental disorders, fourth edition
